# Rational engineering of the *Trichoderma reesei* RUT-C30 strain into an industrially relevant platform for cellulase production

**DOI:** 10.1186/s13068-020-01732-w

**Published:** 2020-05-22

**Authors:** Lucas Miranda Fonseca, Lucas Salera Parreiras, Mario Tyago Murakami

**Affiliations:** Brazilian Biorenewables National Laboratory, Brazilian Center for Research in Energy and Materials, Campinas, São Paulo 13083-100 Brazil

**Keywords:** *Trichoderma reesei*, RUT-C30, CRISPR/Cas9-based genetic engineering, Bioprocess development, Cellulase production

## Abstract

**Background:**

The path for the development of hypersecreting strains of *Trichoderma reesei* capable of producing industrially relevant enzyme titers remains elusive despite over 70 years of research and industrial utilization. Herein, we describe the rational engineering of the publicly available *T. reesei* RUT-C30 strain and a customized process for cellulase production based on agroindustrial by-products.

**Results:**

A CRISPR/Cas9 system was used to introduce six genetic modifications in RUT-C30. Implemented changes included the constitutive expression of a mutated allele of the cellulase master regulator XYR1, the expression of two heterologous enzymes, the β-glucosidase CEL3A from *Talaromyces emersonii* and the invertase SUC1 from *Aspergillus niger*, and the deletion of genes encoding the cellulase repressor ACE1 and the extracellular proteases SLP1 and PEP1. These alterations resulted in a remarkable increase of protein secretion rates by RUT-C30 and amended its well described β-glucosidase deficiency while enabling the utilization of sucrose and eliminating the requirement of inducing sugars for enzyme production. With a developed sugarcane molasses-based bioprocess, the engineered strain reached an extracellular protein titer of 80.6 g L^−1^ (0.24 g L^−1^ h^−1^), which is the highest experimentally supported titer so far reported for *T. reesei*. The produced enzyme cocktail displayed increased levels of cellulase and hemicellulase activities, with particularly large increments being observed for the specific activities of β-glucosidase (72-fold) and xylanase (42-fold). Notably, it also exhibited a saccharification efficiency similar to that of a commercially available cellulase preparation in the deconstruction of industrially pretreated sugarcane straw.

**Conclusion:**

This work demonstrates the rational steps for the development of a cellulase hyperproducing strain from a well-characterized genetic background available in the public domain, the RUT-C30, associated with an industrially relevant bioprocess, paving new perspectives for *Trichoderma* research on cellulase production.

## Background

Large-scale enzyme production technologies are a crucial asset for the development of sustainable bioeconomies. The progressive replacement of traditional chemical catalysts by enzymes and an expanding range of applications drive a growing demand for these environment-friendly biocatalysts [1–3]. Among the emerging industrial enzyme applications, the use of cellulases to obtain fermentable sugars from lignocellulosic biomasses stands out as a promising alternative towards low-carbon and renewable technologies. The cost-efficient enzymatic deconstruction of non-edible lignocellulosic materials, mainly agroindustrial residues, is expected to enable the use of this large and mostly untapped source of carbon for the production of biofuels and value-added chemicals [4–8].

As with other classes of enzymes, the industrial production of cellulases is largely controlled by a few long-established companies [9, 10]. This market dominance arises from, among other factors, the use of proprietary microbial platforms and production processes which have been continuously developed for decades. Industrial strains of the ascomycete fungus *Trichoderma reesei* are among the main platforms employed for enzyme manufacturing, accounting for the production of up to 80% of enzyme formulations currently utilized in the lignocellulosic biofuels industry [11, 12]. *T. reesei*’s predominance in this sector stems from its innate ability to efficiently break down lignocellulose through the coordinated secretion of an unexpectedly small set of carbohydrate active enzymes (CAZymes). Although not fully comprehended, the formidable action of these enzymes is believed to be favored by the fungus’ diverse membrane-trafficking system and clustered genome organization [13].

All industrial *T. reesei* strains and most strains used in academic research nowadays originate from *T. reesei* isolate QM6a, which was originally isolated in the Solomon Islands during World War II [14]. Starting in the early 1970s, random mutagenesis techniques were used to develop several hypercellulolytic mutants of QM6a [15–17]. One of these strains, the RUT-C30, has been intensively studied in academic research and used for the industrial production of cellulases [18, 19]. Available in the public domain and with a well-characterized genetic background, RUT-C30 presents a large 85-kb chromosomal deletion and a truncated *cre1* gene, among a series of other mutations compared to QM6a [20–22]. *Cre1* encodes a sequence-specific DNA-binding protein that mediates carbon catabolite repression (CCR) of cellulase genes by glucose in *T. reesei*. As a result of the *cre1* truncation, RUT-C30 presents a partial relief of CCR by glucose, being capable of reaching extracellular protein titers of up to 20 g L^−1^ [18, 23, 24]. Highly developed industrial strains of *T. reesei* have been reported to reach cellulase titers in excess of 100 g L^−1^ [25]. Nevertheless, experimental data regarding the development of these strains, enzyme productivity or the production processes employed to reach such titers remain mostly unavailable to the scientific community. In general, the enzyme production capacity of *T. reesei* strains used in academic research falls far below that ascribed to industrial variants. For instance, in a study where the proprietary strain VTT-M44 was engineered for enhanced cellulase production, an enzyme titer of 37.3 g L^−1^ (0.20 g L^−1^ h^−1^) was reached using a low-cost process [26]. This is one of the highest experimentally supported protein titers so far described for *T. reesei*. Despite major achievements in the molecular understanding of factors controlling enzyme production in this fungus, the development of strains capable of reaching industrially relevant levels of cellulase production still poses a challenge.

Several genetic modifications have been associated with increased cellulase production by *T. reesei* (reviewed in [12, 27, 28]). For instance, the transcription factor XYR1 has been identified as the major regulator of cellulase-encoding genes in this filamentous fungus and its constitutive expression has been shown not only to increase overall enzyme production, but also to relieve CCR by glucose [29, 30]. Further CCR relief has been achieved with the introduction of individual point mutations (V821F and A824V) in a conserved regulatory region of XYR1 [26, 31]. These mutations are thought to lead to changes in the secondary structure of the protein, causing a “glucose-blind” cellulase-expressing phenotype. Moreover, when the constitutive expression of *xyr1* was combined with the RNA interference (RNAi)-mediated silencing of *ace1*, one of the main repressors of cellulase gene expression in *T. reesei*, even greater improvements in enzyme production were achieved [30, 32]. Other identified regulators of cellulase expression include the activators ACE2 [33], ACE3 [34], the HAP2/3/5 complex [35] and the putative methyltransferase LAE1 [36]. Some modifications that do not target the transcript levels of cellulase-encoding genes have also shown positive effects on cellulase production. A prime example would be the removal of extracellular proteases such as SLP1, a subtilisin protease, and PEP1, which is the most abundant aspartic protease found in *T. reesei* secretome. The deletion of the corresponding *slp1* and *pep1* genes and of other protease-encoding genes has been associated with increased protein secretion rates [37]. Numerous modifications aimed at improving the quality of the enzyme cocktail secreted by *T. reesei* have also been reported. The implementation of lacking enzymatic activities through the expression of heterologous enzymes such as lytic polysaccharide monooxygenases (LPMOs) [38] and ligninases [39] has been shown to improve the saccharification efficiency of the produced cocktail. Most often, however, studies have focused on increasing its β-glucosidase activity, which is the most pronounced and long-studied deficiency of *T. reesei* secretome [40, 41].

Culture medium components are estimated to account for up to 60% of overall enzyme production costs, with the utilized carbon source being by far the most expensive item [42]. Therefore, significant efforts have also been made towards genetically modifying *T. reesei* in order to diversify the range of carbon sources that it can utilize for enzyme production. For instance, molasses derived from sugar manufacturing processes are inexpensive, soluble, highly concentrated and packed with vitamins and minerals, being often considered as a suitable carbon source for enzyme production [26, 43, 44]. Besides high contents of fructose and glucose, sugarcane molasses contain approximately 35% (w/w) of sucrose, representing about 65% of the total available sugar [45]. *T. reesei* lacks invertase activity as a typical secondary colonizer, thus being unable to utilize sucrose or sucrose-containing molasses [13, 46]. This issue has been addressed in previous studies through the engineering of *T. reesei* with the *Aspergillus niger suc1* gene, which encodes an extracellular invertase [26, 46].

In spite of all the above-mentioned genetic modifications known to in some way improve the cellulase production capabilities of *T. reesei*, in only a few instances their combined effects have been investigated. This has been partly due to technical difficulties associated with the genetic engineering of this fungus, such as a reduced number of available selectable markers and low transformation efficiencies [47]. Most of these difficulties have been successfully circumvented in recent years with the use of CRISPR/Cas9 systems [48–52]. The advent of this technology has enabled the efficient and precise insertion of DNA cassettes into the genome of *T. reesei*, greatly facilitating the introduction of multiple modifications in a single strain.

Here, we report the CRISPR/Cas9-based rational engineering of the RUT-C30 strain and the development of an industrially compatible process for cellulase production based on agroindustrial by-products. The specific objectives of the present study were: (1) to enhance the β-glucosidase activity of the produced cocktail; (2) to increase protein secretion rates; (3) to eliminate the need of inducing sugars for enzyme production; (4) to enable the use of sucrose-rich molasses as a carbon source; and (5) to develop a molasses-based enzyme production process. Employing the developed strain and bioprocess, an extracellular protein titer of 80.6 g L^−1^ (0.24 g L^−1^ h^−1^) was achieved with high levels of cellulase and hemicellulase activities. The rational design of a publicly available *T. reesei* strain into a hyperproducing platform along with a simple bioprocess based on a low-cost carbon source represents an important step towards new alternatives for industrial cellulase production. The work here described might also impact further research in *Trichoderma*, setting a new standard for the systematic and combinatorial study of other known factors.

## Results

### Rational engineering of *T. reesei* RUT-C30

**Fig. 1:**
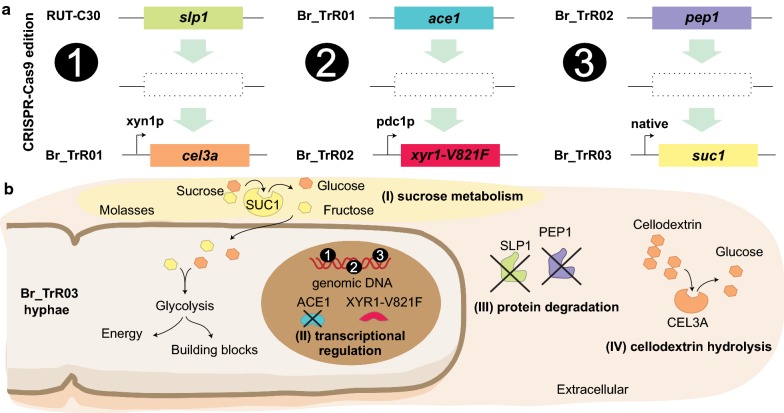
Genetic engineering of the RUT-C30 strain for improved cellulase production. **a** A total of six genetic changes were introduced in RUT-C30 through three rounds of engineering using a CRISPR/Cas9 system. In round 1, the *T. emersonii cel3a* gene under control of the *xyn1* promoter was inserted in the *slp1* locus. In round 2, the mutated *xyr1*-V821F allele under control of the *pdc1* promoter was inserted in the *ace1* locus. In round 3, the *A. niger suc1* gene driven by its native regulatory sequences was inserted in the *pep1* locus. **b** The implemented modifications aimed to improve the fungus enzyme production capabilities by (I) enabling the use of sucrose as a carbon source, (II) increasing transcription levels of genes encoding secreted enzymes, (III) decreasing the proteolytic activity of its secretome and (IV) increasing the β-glucosidase activity of the secreted enzyme cocktail

To efficiently perform iterative cycles of genetic engineering, a single-plasmid CRISPR/Cas9 system and markerless donor cassettes were employed. Target-specific CRISPR/Cas9 plasmids carried a *Streptococcus pyogenes* Cas9 gene codon-optimized for expression in *T. reesei*, a ribozyme-mediated guide RNA (gRNA) cassette, an antibiotic resistance selection marker and the AMA1 fungal replicator sequence (Additional file 1: Fig. S3) [53]. Using this system, six genetic changes were implemented in RUT-C30 through three rounds of engineering (Fig. 1).

**Fig. 2:**
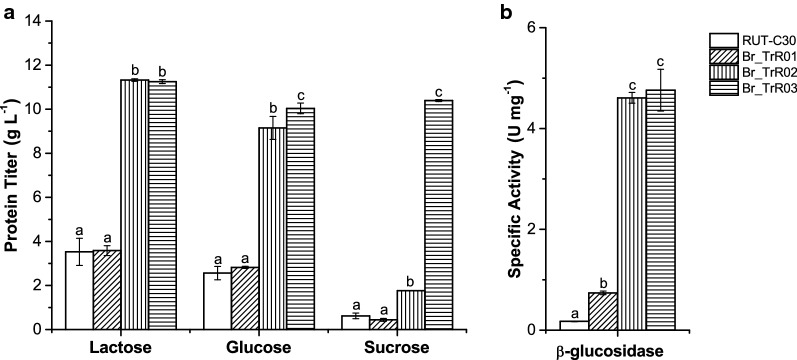
Extracellular protein production in shake flasks with selected carbon sources. The parental RUT-C30 and the engineered strains were cultivated for 5 days in shake flasks with medium containing 50 g L^−1^ of lactose, glucose or sucrose. **a** Extracellular protein concentrations measured from culture supernatants. **b** β-Glucosidase activity from culture supernatants of each strain grown in lactose-containing medium. Data are expressed as mean ± SD (*n* = 3). Tukey post hoc tests were performed (*P* < 0.05) and bars sharing the same letter do not present statistically significant differences

In the first round of modifications, the β-glucosidase-encoding gene *cel3a* from *Talaromyces emersonii* under the control of the xylanase 1 (*xyn1*) promoter was inserted in the locus of the subtilisin protease-encoding gene *slp1* [26, 54, 55]. The generated strain, BR_TrR01, yielded significantly higher levels of β-glucosidase activity than the parental RUT-C30 strain, indicating the functional expression of the *cel3a* gene. As depicted in Fig. 2, the specific β-glucosidase activity presented by BR_TrR01 (0.74 ± 0.03 IU mg^−1^) was about fourfold greater than the detected for RUT-C30 (0.18 ± 0.01 IU mg^−1^) when grown in shake flasks containing lactose medium. The deletion of the *slp1* gene, however, did not lead to significant changes in protein secretion under these same conditions.

Having achieved a significant increase in β-glucosidase activity with the implemented modifications, we turned our focus to genetic changes that could potentially increase enzyme production and abolish glucose-induced CCR, reducing the need for inducing sugars. In line with this objective, the *ace1* gene in the BR_TrR01 strain was replaced by a mutated *xyr1* allele (V821F) driven by the pyruvate decarboxylase 1 (*pdc1*) constitutive promoter (Fig. 1) [26, 56]. The resulting strain, BR_TrR02, displayed a remarkable threefold increment in extracellular protein titer as compared to Br_TrR01. When grown in shake flasks containing lactose medium, Br_TrR01 reached a protein concentration of 3.6 (± 0.2) g L^−1^ while Br_TrR02 yielded 11.3 (± 0.1) g L^−1^ (Fig. 2a). Notably, a similar increase in enzyme production was observed when these strains were cultivated with CCR-inducing glucose. Under these conditions, Br_TrR01 and Br_TrR02 produced 2.8 (± 0.1) and 9.2 (± 0.5) g L^−1^ of extracellular proteins, respectively. The implemented genetic changes also led to a further enhancement in the β-glucosidase activity of the secreted enzyme cocktail. In lactose medium, Br_TrR02 exhibited a specific β-glucosidase activity of 4.6 (± 0.1) IU mg^−1^, representing increases of approximately sixfold when compared to Br_TrR01 and 26-fold compared to the parental RUT-C30 (Fig. 2b).

Next, a third round of modifications was performed with the aim of enabling the utilization of sucrose and to further augment cellulase production by the engineered strain. To this purpose, the invertase-encoding gene *suc1* from *Aspergillus niger* flanked by its native regulatory sequences was inserted in the genome of Br_TrR02, replacing the *pep1* gene (Fig. 1). The extracellular protein titer achieved with the newly generated strain, named Br_TrR03, was similar to that of Br_TrR02 when grown in shake flasks containing lactose (Fig. 2a). A slight increase in protein production was noted for Br_TrR03 when the two strains were cultivated in glucose medium. Importantly, Br_TrR03 also reached comparable enzyme titers when grown in sucrose-containing medium. Unable to consume sucrose (Additional file 1: Fig. S1), the parental RUT-C30 and the intermediate strains Br_TrR01 and Br_TrR02 produced minimal amounts of extracellular proteins under these conditions (Fig. 2a).

**Fig. 3:**
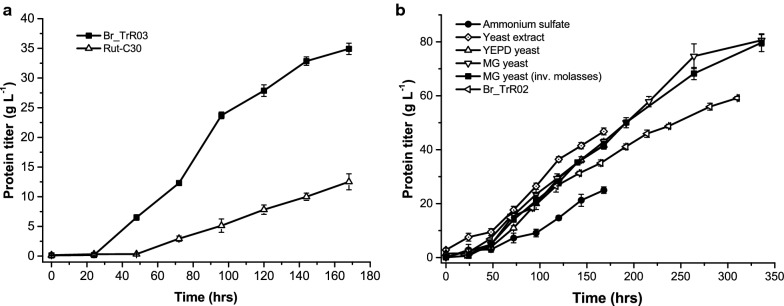
Extracellular protein production in bioreactors under fed-batch mode. **a** Comparison between RUT-C30 and the engineered Br_TrR03 strain in bioreactor cultivations with an inducer-rich medium. **b** Extracellular protein production by Br_TrR03 during bioreactor cultivations with a molasses-based batch medium supplemented with different nitrogen sources: ammonium sulfate only, yeast extract, YEPD-grown yeast (YEPD yeast) and molasses-grown yeast (MG-yeast). The feed comprised sugarcane molasses or acid-inverted molasses (inv. molasses). For comparison purposes, a fermentation with the strain Br_TrR02 using acid-inverted molasses and molasses-grown yeast is shown. Results are expressed as mean ± SD (*n* = 3)

To better evaluate the effects of the implemented genetic changes on cellulase production, RUT-C30 and Br_TrR03 were cultivated in bioreactors with an inducer-rich medium. Fermentations were conducted under fed-batch mode with a batch medium containing the inducers lactose and microcrystalline cellulose (Avicel) and a feed that comprised lactose and glucose. Yeast extract was added to the batch medium as an organic nitrogen source. During trial experiments, it was noticed that dissolved oxygen concentrations could be maintained at set values with higher sugar feed rates for Br_TrR03 than for RUT-C30. In order to make the comparison under the exact same conditions, sugars were fed to both strains at the rate which enabled maximum protein production with the parental strain. Under these conditions, RUT-C30 produced 12.5 g L^−1^ of extracellular proteins in 168 h (0.07 g L^−1^ h^−1^), while BR_TrR03 reached a protein concentration of 34.9 g L^−1^ (0.21 g L^−1^ h^−1^) (Fig. 3a). These results confirmed a near threefold increase in enzyme productivity for Br_TrR03 compared to RUT-C30. Still, some of the medium components used in the performed fermentations, such as lactose and Avicel, were very costly, making the application of this process on an industrial scale highly unlikely. As such, we set out to develop a process for cellulase production with the engineered BR_TrR03 strain based on inexpensive and sugar-rich sugarcane molasses.

### Sugarcane molasses-based cellulase production process

In a first attempt to utilize sugarcane molasses as a carbon source for cellulase production with Br_TrR03, a fed-batch bioreactor fermentation was conducted with a feed that comprised only molasses and a batch medium containing molasses and ammonium sulfate. This fermentation resulted in an extracellular protein concentration of 25.0 g L^−1^ in 168 h (0.15 g L^−1^ h^−1^) (Fig. 3b), which corresponds to approximately 70% of the titer obtained with the inducer-rich medium. Based on the reasoning that the observed decrease in productivity might be due to the absence of an organic nitrogen source, the experiment was repeated with the addition of yeast extract to the batch medium, what led to a remarkable gain in enzyme productivity (Fig. 3b). With a feed that comprised molasses and a batch phase medium made up of molasses, ammonium sulfate and yeast extract, Br_TrR03 reached an extracellular protein concentration of 46.8 g L^−1^ in 168 h (0.28 g L^−1^ h^−1^), surpassing the results obtained with the inducers lactose and Avicel.

Considering the high cost of yeast extract, we sought to determine if molasses-grown yeast could be used to replace it in the batch phase medium. In an initial test, yeast cells were propagated in rich YEPD (yeast extract peptone dextrose) lab medium, washed and then added to the fungal batch medium. The replacement of yeast extract with YEPD-grown yeast cells resulted in a decrease in productivity of approximately 10% (Fig. 3b), with Br_TrR03 producing 41.7 g L^−1^ of extracellular proteins in 168 h (0.25 g L^−1^ h^−1^). Despite the decreased productivity, the obtained results demonstrated the viability of using whole yeast cells as an organic nitrogen source in the fungal medium. We therefore proceeded to test the use of non-washed molasses-grown yeast cells as a replacement for yeast extract. In this experiment, yeast cells were propagated in a standard molasses and salts medium, centrifuged and then added directly to the batch medium. With the molasses-grown yeast, Br_TrR03 reached an extracellular protein concentration of 42.9 g L^−1^ in 168 h (0.26 g L^−1^ h^−1^), a result similar to that achieved with YEPD-grown yeast (Fig. 3b). This fermentation was extended to 336 h (14 days), time at which Br_TrR03 reached a protein titer of 80.6 g L^−1^ with an overall production rate of 0.24 g L^−1^ h^−1^.

Given that no major differences in protein titers were observed between Br_TrR02 and Br_TrR03 in the conducted shake flask experiments, we aimed to determine whether only the 4 genetic modifications carried by Br_TrR02 would suffice to reach the high enzyme concentrations produced by Br_TrR03 in bioreactors. To address this question, Br_TrR02 was cultivated in a bioreactor with the medium containing sugarcane molasses and molasses-grown yeast (MMGY medium). Due to a lack of invertase activity by Br_TrR02, the sucrose in the molasses was inverted by acid hydrolysis prior to the experiment. In an initial fermentation it was determined that Br_TrR02 could not be cultivated with a sugar feed rate as high as the one used for Br_TrR03. Under these conditions, it was not possible to maintain the dissolved oxygen concentration at set values, as Br_TrR02 generated a more viscous culture broth. The feed rate was then optimized for this strain and the experiment repeated. Under these adjusted conditions, Br_TrR02 displayed an enzyme production rate considerably lower than that of Br_TrR03, reaching a protein titer of 59.2 g L^−1^ in 310 h (0.19 g L^−1^ h^−1^) (Fig. 3b). This experiment still left open the possibility that the acid hydrolysis of molasses impacted its nutritional properties, affecting the performance of Br_TrR02. To address this concern, Br_TrR03 was cultivated in MMGY medium containing acid-inverted molasses. As depicted in Fig. 3b, Br_TrR03 displayed an almost identical performance to that exhibited with the non-inverted molasses, reaching a protein titer of 79.7 g L^−1^ in 336 h (0.24 g L^−1^ h^−1^). Lastly, to further validate the obtained results, the Br_TrR02 strain was reconstructed starting from a RUT-C30 stock acquired from a different collection (Br_TrR02B). When cultivated in bioreactors containing the MMGY medium, Br_TrR02 and Br_TrR02B presented virtually identical enzyme production rates (Additional file 1: Fig. S2).

### Enzyme activity profiling

To assess the saccharolytic potential of the enzyme cocktails produced by Br_TrR03 and RUT-C30, cellulolytic and hemicellulolytic activities were profiled. The analyzed cocktails were those produced by Br_TrR03 and RUT-C30 in bioreactor fermentations with the inducer-rich medium and by Br_TrR03 with the MMGY medium. Cellulolytic activity tests included cellobiohydrolase, filter paper activity (FPase), endoglucanase and β-glucosidase, while the assessed hemicellulolytic activities were xylanase and β-xylosidase.

**Fig. 4:**
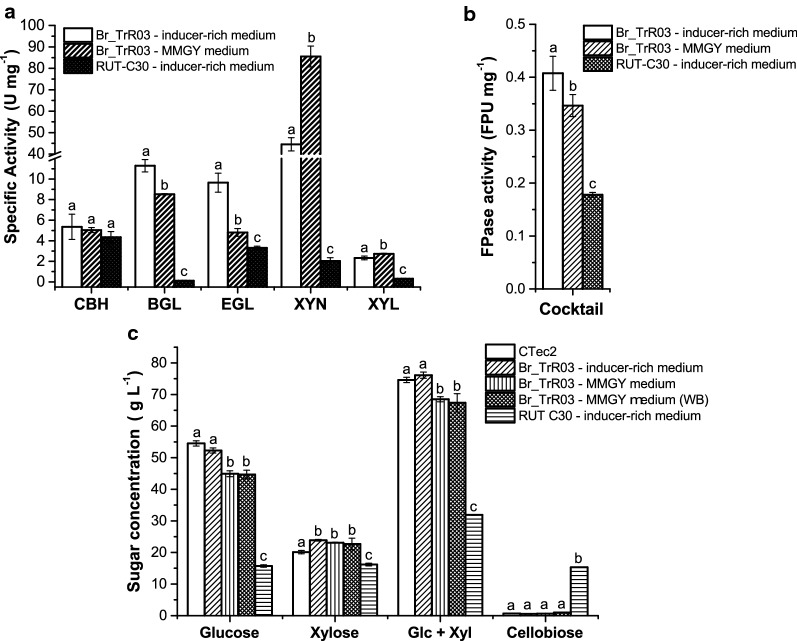
Characterization of enzyme cocktails produced in bioreactors. Specific activity levels of **a** cellobiohydrolase (CBH), β-glucosidase (BGL), endoglucanase (EGL), xylanase (XYN) and β-xylosidase (XYL). **b** Filter paper activity. **c** Saccharification efficiencies on industrially pretreated sugarcane straw. The performance of the whole fermentation broth (WB) from Br_TrR03 cultivated on MMGY medium was also determined. The commercially available CTec2 enzyme cocktail was included as a reference. Results are expressed as mean ± SD (*n* = 3). Tukey post hoc tests were performed (*P* < 0.05) and bars sharing the same letter in each panel do not present significant statistical differences

The genetic changes carried by Br_TrR03 led to significant increments in specific enzymatic activity. Even when cultivated with the industrially relevant MMGY medium, Br_TrR03 yielded higher levels of specific enzymatic activity in 5 out of 6 performed assays, compared to RUT-C30 in the inducer-rich medium (Fig. 4a, b). As a result of the insertion of the gene *cel3a* driven by the *xyn1* promoter, Br_TrR03 displayed an increase in β-glucosidase-specific activity of approximately 72-fold. The second largest increment was observed for the specific activity of xylanase, which was about 42-fold greater for the Br_TrR03-MMGY cocktail than for the RUT-C30 cocktail. Of note, when cultivated in the inducer-rich medium, Br_TrR03 displayed a further increase in cellulolytic activities, indicating that the fermentation medium still affects the secretome of the engineered strain.

The increased enzymatic activities observed for the cocktails produced by Br_TrR03 suggested that these might perform better than the RUT-C30 cocktail in the saccharification of lignocellulosic biomasses. To assess this possibility, the characterized cocktails were used in the saccharification of industrially pretreated sugarcane straw. To better emulate industrial conditions, saccharification assays were conducted with high solids content (20% w/w). The whole broth (WB) from the Br_TrR03-MMGY fermentation was also tested in this assay. A commercially available cellulase cocktail derived from *T. reesei,* CTec2, was included as a reference control.

As shown in Fig. 4c, both Br_TrR03 cocktails yielded more glucose, xylose and overall more sugars than the RUT-C30 cocktail. Also, saccharification of the pretreated biomass with Br_TrR03 cocktails did not result in cellobiose accumulation, as opposed to the observed for RUT-C30. It was also noted that the addition of the whole broth from the Br_TrR03-MMGY fermentation did not affect the saccharification efficiency of the cocktail. Moreover, as observed with the enzymatic activity levels, the medium used for the cultivation of Br_TrR03 had an effect on the saccharification efficiency of the produced cocktail. The enzymes secreted by Br_TrR03 in the MMGY medium released slightly less glucose and overall less sugars than those secreted in the inducer-rich medium. The saccharification performance of the Br_TrR03 cocktail produced with the inducer-rich medium was equivalent to that of the enzyme cocktail CTec2 with regards to released glucose and overall sugar yield.

## Discussion

Since it was first isolated over 70 years ago, *T. reesei* has been intensively studied in both academic and industrial laboratories [11, 12]. Its enzyme secretion capabilities drew the attention of the industry, which set out to develop it into a commercially important enzyme production platform. Despite numerous reports of genetic modifications associated with increased enzyme secretion rates, the path for the development of strains capable of reaching industrially relevant enzyme titers remains unclear. Herein, a single-plasmid CRISPR/Cas9 system and markerless donor cassettes were used to combine six previously described genetic modifications in the prototype hyperproducer strain RUT-C30.

The CRISPR/Cas9 plasmid used in the present study was adapted from a report where a similar plasmid was used to engineer six different species of *Aspergillus* [53]. As described here, this system has also proved efficient in the engineering of a *T. reesei* strain. In our experiments, an overall transformation efficiency ranging from 10 to 16% was observed for the genomic integration of donor cassettes (data not shown). It should be noted that a recent report indicated that the use of CRISPR/Cas9 systems with the intracellular expression of Cas9 may lead to unexpected mutations in *T. reesei* [51]. Even though no evidence of the occurrence of off-target mutations, such as phenotypic variability among clones, was observed during our study, this possibility cannot be ruled out without conducting the whole-genome sequencing of engineered strains.

The modifications introduced in RUT-C30 aimed to improve its cellulase secretion capabilities by focusing on different levels of its enzyme production machinery. The transcription of cellulase-encoding genes was targeted through the simultaneous modification of two major transcription factors, *xyr1* and *ace1*. In a previous report, the constitutive expression of *xyr1* combined with the RNAi-mediated silencing of *ace1* resulted in an increment of 103% in enzyme secretion by RUT-C30 in Avicel medium [30]. This improvement was, however, drastically reduced when the modified strain was grown in glucose. In comparison, the constitutive expression of *xyr1*-V821F and the simultaneous deletion of *ace1* in the engineered Br_TrR02 strain resulted in an increase of about 220% in enzyme production in both lactose and glucose media. The greater enhancements observed in our experiments are likely due to the V821F mutation introduced in XYR1, as large improvements in both enzyme production and CCR relief have also been observed when the *xyr1*-V821F allele was constitutively expressed in a proprietary *T. reesei* strain [26]. Additionally, the presence of the intact wild type *xyr1* allele in the genome of the engineered strain may influence the effect of the constitutive expression of its mutated counterpart. XYR1 can form homodimers when inducing the expression of cellulase genes [57]. The presence of the two forms of XYR1 in the cell nucleus would therefore result in the formation of three types of dimers, each of which may have a different effect on the regulation of cellulase gene expression.

A second strategy employed to augment cellulase production by the engineered strain was to reduce the proteolytic activity of the secreted cocktail through the deletion of protease-encoding genes. Protease deficient fungal strains have been shown to produce greater quantities of extracellular enzymes [37, 58, 59]. It has been speculated that this enzyme-boosting effect may be due to a compensatory response by the fungi to the reduced availability of nutrients from protein breakdown. An additional possibility raised is that the reduced proteolytic activity of the enzyme cocktail increases the stability of secreted native proteins. In the developed Br_TrR03 strain, two of the major proteases found in *T. reesei* secretome, SLP1 and PEP1, were removed. These enzymes have been shown to, respectively, account for 18% and 42% of the total proteolytic activity against casein in the secretome of a proprietary *T. reesei* strain [37]. This same study showed that the deletion of six protease-encoding genes, including *slp1* and *pep1*, reduced in over 93% the proteolytic activity of the secreted cocktail and led to a 22% increase in cellulase production. In shake flask cultivations with equal amounts of sugar available to each of our engineered strains, the deletion of neither *slp1* nor *pep1* had a major effect on cellulase production. In contrast, under controlled bioreactor fermentations, it was observed an increase in enzyme productivity of approximately 24% for Br_TrR03 that carried both *slp1* and *pep1* deletions when compared to Br_TrR02, harboring only the *slp1* modification. This result is likely associated with the higher feed rates enabled by Br_TrR03. Nevertheless, no definitive conclusions about the individual effects of the *slp1* and *pep1* gene deletions can be drawn given that these modifications were performed simultaneously with the insertions of the *cel3a* and *suc1* genes, respectively.

Having achieved a near threefold increase in enzyme productivity for the sucrose-utilizing Br_TrR03 strain compared to the parental RUT-C30 in the inducer-rich medium fermentations, we set out to develop a production process based on sugarcane molasses. The productivity displayed by Br_TrR03 in the rich-medium experiments (0.21 g L^−1^ h^−1^) was not maintained, however, with a batch medium made up of molasses and ammonium sulfate. Based on a previous report of an organic nitrogen source being required for maximum enzyme production by *Trichoderma*, it was reasoned that the addition of yeast extract to the molasses-based medium might improve the observed results [60]. And in fact, when yeast extract was added to the batch medium, Br_TrR03 exhibited an enzyme productivity of 0.28 g L^−1^ h^−1^. This is, to the best of our knowledge, the highest cellulase production rate so far reported for *T*. *reesei*. The highest rate previously described was 0.23 g L^−1^ h^−1^ [61]. This result was achieved in bioreactor cultivations of the M3-1 strain, which was developed from QM6a through multiple rounds of mutagenesis. A highly elaborated medium containing cellobiose, xylose, glucose, yeast extract, peptone and Tween 80, among other components, was used in these experiments with M3-1.

Despite the high enzyme productivity achieved in the fermentation with the sugarcane molasses and yeast extract medium, we sought to find a more suitable source of organic nitrogen. Processed medium components such as yeast extract and peptone have long been considered too expensive for industrial use [60]. An alternative proposed early on was the use of *Trichoderma* mycelium from previous fermentations as a source of organic nitrogen. It was also envisioned that agroindustrial organic residues could potentially be used for this purpose [60]. Examples of residues so far identified as suitable nutrient sources for enzyme production with *Trichoderma* include corn steep liquor, spent brewery grain, soybean husk and soybean meal [26, 62–64]. Here, we have demonstrated the feasibility of using molasses-grown whole yeast cells as an organic nitrogen source for the production of cellulases with *T. reesei*. When cultivated with molasses-grown yeast (MMGY medium), Br_TrR03 presented an enzyme productivity comparable to that achieved with yeast extract, reaching an extracellular protein titer of 80.6 g L^−1^. As far as we are aware, this is the highest cellulase titer so far described in original research for *T. reesei*.

Considering the high protein titers achieved and the inexpensive nature of the MMGY medium components, it seems reasonable to assume that the enzymes produced with Br_TrR03 would have a low overall cost. However, a detailed techno-economic analysis would be required to accurately assess the cost-competitiveness of the produced cellulase cocktail. In addition to a potentially low cost, the MMGY medium brings the added advantage of a reduced number of medium components that must be outsourced. As with other manufactured goods, fluctuations in quality and availability of raw materials may directly impact the properties and price of the finished enzyme product [11]. The use of molasses to generate yeast cells would remove the need for the additional outsourcing of a suitable organic nitrogen source. The approach of using whole yeast cells as nutrient for enzyme production may be especially appealing to lignocellulosic and corn-based ethanol factories which routinely cultivate yeast and have a demand for large quantities of enzymes.

Besides displaying increased production rates, the engineered Br_TrR03 strain produced a cellulase cocktail with higher specific enzymatic activities and significantly better saccharification efficiencies than the parental RUT-C30. The major improvement in β-glucosidase activity is likely due to both the insertion of the *cel3a* gene, driven by the *xyn1* promoter, and the constitutive expression of the transcription factor *xyr1.* The constitutive expression of *xyr1* is known to particularly increase transcription levels of hemicellulase-encoding genes, including *xyn1* [29]. This would also help to explain the fact that a considerably greater increment was observed for the specific activity of xylanase as compared to cellulolytic activities, such as cellobiohydrolase and endoglucanase. Greater enhancements of hemicellulolytic activities were also observed in a previous study where a proprietary *T. reesei* strain was engineered with the same *xyr1*-V821F allele as Br_TrR03 [26]. The effectiveness of the enhanced β-glucosidase activity exhibited by our engineered strain was evidenced in the performed saccharification assay with an industrially pretreated biomass at high solids content. While deconstruction of the pretreated biomass with the RUT-C30 enzyme cocktail led to the accumulation of cellobiose, minimal quantities of this disaccharide were detected with Br_TrR03 cocktails, which presented a saccharification performance similar to that of the commercial cellulase preparation CTec2. Also, the addition of the whole fermentation broth from the Br_TrR03-MMGY fermentation did not affect the saccharification efficiency of the cocktail. In the scenario of on-site manufacturing (OSM) of cellulases, the use of unprocessed whole fermentation broths has been proposed as a cost-reducing alternative for lignocellulosic biorefineries as it would avoid transportation, storage and downstream processing-related expenses [65–67].

Differences in enzymatic activity and saccharification efficiencies were also noted between the enzyme cocktails secreted by Br_TrR03 in different media. These results indicate that although the newly developed strain does not require inducing sugars for efficient cellulase production, the profile of the secreted cocktail is still affected by the medium composition. The understanding of the molecular basis of the observed differences, possibly through transcriptome and secretome studies, may reveal candidate gene targets and help guide the further development of the fungal platform. Additional improvements in the saccharification efficiency of the produced cocktail may also be achieved through the implementation of lacking enzymatic activities. The same is true for the enzyme productivity displayed by the fungus, which might be improved through the modification of additional transcription factors and protease-encoding genes.

## Conclusion

In this work, we describe the development of the publicly available *T. reesei* RUT-C30 strain into an industrially relevant cellulase production platform. The implementation of a minimal set of six genetic modifications combined with a developed sugarcane molasses-based process enabled the achievement of a cellulase titer of 80.6 g L^−1^ (0.24 g L^−1^ h^−1^), which is the highest experimentally supported titer so far reported for *T. reesei*. In addition, the saccharification efficiency of the produced enzyme cocktail was found to be similar to that of a commercially available cellulase preparation. Notably, this work can be reproduced by other research groups as RUT-C30 is readily available through various strain collections, in contrast to studies based on proprietary strains.

## Materials and methods

### Growth and maintenance of fungal strains

*Trichoderma reesei* RUT-C30 (IHEM_5652/ATCC_56765) and derived engineered strains were grown on Potato Dextrose Agar (PDA; Merck, Darmstadt, Germany) plates at 28 °C until conidiation was observed (5–10 days). Conidia spores were suspended in a 20% *(v/v)* glycerol solution, filtered through sterile cotton for the removal of hyphae, quantified with a hemocytometer and stored at − 80 °C.

### *Trichoderma reesei* CRISPR/Cas9 system and donor cassettes

All oligonucleotides used in this study are listed in Additional file 1: Table S1. Plasmids were propagated in *E. coli* DH5α strain and purified with the QIAprep Miniprep kit (Qiagen, Hilden, Germany). PCR amplifications were performed with Phusion high-fidelity DNA polymerase following the manufacturer’s instructions (NEB, Ipswich, USA). PCR products and DNA restriction fragments were purified with QIAquick kits (Qiagen). Sanger DNA sequencing was conducted with an Applied Biosystems (Foster City, USA) 3500xL Genetic Analyzer.

The CRISPR/Cas9 base vector, plasmid pTrCas9gRNA1 (GenBank MN555456), was assembled through the stepwise ligation of 6 linear DNA fragments containing 4 bp overhangs: (i) AMA1 replicator, (ii) Ppdc1_ToCas9_Tpdc1, (iii) Peno1, (iv) Txyn1, (v) *hph* cassette and (vi) pUC19 backbone. The AMA1 fungal plasmid replicator fragment was excised from the ANEp7 vector through NotI-HF (NEB) digestion and agarose gel purification [68, 69]. The remaining 5 fragments were PCR-amplified with primers containing BsaI restriction sites. If required, internal BsaI sites were removed by overlap extension PCR or during the design of purchased constructs. The Ppdc1_ToCas9_Tpdc1 fragment was amplified from a construct containing the Cas9 gene from *Streptococcus pyogenes* with a SV40 nuclear localization signal, codon-optimized for expression in *T. reesei* [49] and flanked by the *pdc1* gene promoter and terminator sequences (supplied by GenScript, Piscataway, USA). The *eno1* promoter [56] and *xyn1* terminator fragments were amplified from genomic DNA of strain IHEM_5652. The *hph* cassette [70] and the pUC19 backbone [71] fragments were amplified from pre-existing plasmids. PCR products were gel-purified, digested with BsaI-HF (NEB) and then column-purified. Adjacent fragments (ii and iii; iv and v; i and vi) were first ligated pairwise with T7 DNA ligase (NEB) at 25 °C for 4 h. The 3 ligation reactions were then combined and incubated at 25 °C for another 4 h before electroporation into *E. coli*. Pairs of internal primers specific to the ends of each DNA fragment were used to screen obtained transformants by colony PCR. Correct assembly of the plasmid was further confirmed through Sanger sequencing. A Bsp1407I restriction site located between the *eno1* promoter and the *xyn1* terminator in the pTrCas9gRNA1 plasmid was used to generate CRISPR/Cas9 vectors for targeting of specific *T. reesei* genes. For each target gene, a DNA fragment containing a chimeric guide RNAs (gRNA) sequence flanked by (5′-end) hammerhead (HH) and (3′-end) hepatitis delta virus (HDV) ribozyme sequences was generated and inserted into the Bsp1407I site (GenBank MN555452–MN555454) [53, 72]. These insert fragments were generated by assembly PCR of two ssDNA oligonucleotides (98 bp and 158 bp) with a 23 bp overlap and subsequent digestion with FastDigest Bsp1407I (Thermo Scientific, Waltham, USA). Correct insertion of each fragment in the Bsp1407I-digested pTrCas9gRNA1 plasmid was verified by colony PCR and Sanger sequencing. The 20-nt protospacers designed to target-specific genes in *T. reesei* are highlighted (bold characters) within the corresponding oligo sequences in Additional file 1: Table S1.

Markerless donor cassettes for genomic integration were assembled on plasmids by in vivo homologous recombination in *Saccharomyces cerevisiae*, as previously described [73]. DNA fragments containing 40 bp overlap sequences were PCR-amplified from fungal genomic DNA and co-transformed into *S. cerevisiae* strain PE-2 [74] with pRS315 plasmid backbone and kanMX cassette PCR fragments [75, 76]. Each cassette contained 1 kb flanking sequences homologous to regions upstream and downstream of the *T. reesei* gene targeted for deletion. All *T. reesei* genome fragments were amplified from strain IHEM_5652. The *xyr1*-V821F fragment was generated by overlap extension PCR with a pair of internal primers containing the desired mutation. The beta-glucosidase-encoding gene *cel3a* was amplified from gDNA of a *Talaromyces emersonii* strain recently isolated in the Campinas area, Brazil, and kindly provided by Dr. Roberto Ruller (manuscript in preparation). The invertase-encoding gene *suc1* and its flanking regulatory sequences were amplified from the genome of *Aspergillus niger* N402 strain (FGSC_A733) [77]. The sequences of plasmids containing the cassettes (i) Ppdc1_xyr1-V821F_Tpdc1:ace1Δ, (ii) Pxyn1_TeCel3a_Txyn1:slp1Δ and (iii) AnSuc1:pep1Δ were deposited in GenBank under accession numbers MN555457-MN555459. Each cassette was PCR-amplified from the respective plasmid to generate linear DNA fragments used for fungal transformation. Obtained PCR products were column-purified and concentrated in a Speed-Vac concentrator prior to use in transformations.

### Protoplast transformation

*Trichoderma reesei* protoplasts were generated and transformed as described by Penttila et al. [78]. In each transformation event, 3–4 μg of the appropriate CRISPR/Cas9 vector and 2–5 μg of a linear DNA fragment for genomic integration were used. Transformants were selected by plating on minimal media containing 15.0 g L^−1^ KH_2_PO_4_, 5.0 g L^−1^ (NH_4_)_2_SO_4_, 0.59 g L^−1^ MgSO_4_, 0.45 g L^−1^ CaCl_2_, 5.0 mg L^−1^ FeSO_4_·7H_2_O, 2.0 mg L^−1^ CoCl_2_·6H_2_O, 1.6 mg L^−1^ MnSO_4_·4H_2_O, 1.4 mg L^−1^ ZnSO_4_·7H_2_O, 1 M sorbitol, 20.0 g L^−1^ agar and 100 μg mL^−1^ of hygromycin B (Merck). The medium pH was adjusted to 5.5 with 3 M KOH. Transformation plates were incubated at 28 °C for 6–10 days until colonies were observed. Obtained colonies were transferred to PDA plates and allowed to grow for 7–10 days at 28 °C until conidiation. Conidia spores were suspended in 500–1000 μL of dH_2_O, inoculated into 5 mL of liquid potato dextrose (PD) medium (Sigma, St. Louis, USA) and incubated at 28 °C with 200 rpm for 72 h in order to generate enough hyphae for DNA extraction. These same spore suspensions were streaked on 0.1% *(v/v)* Triton X-100 PDA plates for spore isolation and incubated at 28 °C for 72 h. DNA extractions were performed with the Quick-DNA Fungal/Bacterial Miniprep Kit from Zymo Research (Irvine, USA). PCR verifications were performed with different combinations of primers that anneal (up/down)stream of the targeted genome regions or internally to each integration cassette (Additional file 1: Figs. S4–S7). Germinated spores from verified transformants were transferred from the PDA/Triton X-100 plates to fresh PDA media for growth and conidiation. Each isolate was subjected to at least two additional rounds of DNA extraction, PCR verification and spore isolation in order to obtain stable, homokaryotic and marker-free transformants. The three genetic modifications carried by the final strain (Br_TrR03) were further verified through Sanger sequencing of PCR amplicons which spanned the entire modified loci (generated with P1/P4 primer sets). Loss of the CRISPR/Cas9 vector during conidiation was confirmed by the streaking of isolates on minimal media plates containing 100 μg mL^−1^ of hygromycin B, as previously described [53].

### Culture medium components

Sugarcane molasses with a total sugar concentration of approximately 740 g L^−1^ were provided by Mellaço de Cana (Saltinho, Brazil). When required, the sucrose contained in the molasses was inverted by first diluting it with water to a total sugar concentration of approximately 600 g L^−1^. The pH of the solution was lowered to 2.2 with concentrated hydrochloric acid and it was then autoclaved using a standard liquid cycle.

Whole yeast cell preparations were generated by inoculating colonies of *S. cerevisiae* strain PE-2 [74] grown on yeast peptone (YP) 2% glucose agar plates into 500 mL of liquid medium in 2-L Erlenmeyer flasks and incubation at 30 °C with 200 rpm for 48 h. For the molasses-grown yeast preparation, a medium that comprised 1.0 g L^−1^ (NH_4_)_2_HPO_4_, 2.2 g L^−1^ MgSO_4_, 2.0 g L^−1^ ZnSO_4_·7H_2_O and 110 g L^−1^ of sugarcane molasses was used for cultivation. Yeast cells were centrifuged at 10,000×*g* for 10 min and added directly to the fungal medium. For the YEPD-grown yeast preparation, YP 7.5% glucose medium was used. Yeast cells were centrifuged at 10,000×*g* for 10 min and washed three times with 500 mL of sterile dH_2_O prior to its addition to fungal media. The dry mass of yeast cell pellets was determined with a Shimadzu (Kyoto, Japan) MOC63u moisture analyzer.

### Shake flask and bioreactor fermentations

*Trichoderma reesei* strains were grown in 500-mL Erlenmeyer flasks containing 100 mL of medium that comprised 10.0 g L^−1^ yeast extract, 15.0 g L^−1^ KH_2_PO_4_, 10.0 g L^−1^ (NH_4_)_2_SO_4_, 0.59 g L^−1^ MgSO_4_, 0.45 g L^−1^ CaCl_2_, 5.0 mg L^−1^ FeSO_4_·7H_2_O, 2.0 mg L^−1^ CoCl_2_·6H_2_O, 1.6 mg L^−1^ MnSO_4_·4H_2_O, 1.4 mg L^−1^ ZnSO_4_·7H_2_O, 50 mM Na^−^ succinate buffer, 50.0 g L^−1^ of a soluble carbon source (lactose, glucose or sucrose) and an NaOH-adjusted pH of 4.8. Each flask was inoculated with 5 × 10^6^ spores and cultivations were carried out at 28 °C with 200 rpm for 5 days. Following this period, samples were collected, centrifuged at 14,000×*g* for 10 min and the supernatants were stored at − 20 °C until analysis.

Bioreactor experiments were conducted with BioFlo/CelliGen 115 systems (Eppendorf, Hamburg, Germany) and water-jacketed 3.0 L vessels with a working volume of 1.0–1.5 L. The batch composition for inducer-rich medium fermentations was 20.0 g L^−1^ Avicel PH-101, 10.0 g L^−1^ Lactose, 20.0 g L^−1^ yeast extract, 20.0 g L^−1^ (NH_4_)_2_SO_4_, 15.0 g L^−1^ KH_2_PO_4_, 0.59 g L^−1^ MgSO_4_, 0.45 g L^−1^ CaCl_2_, 5.0 mg L^−1^ FeSO_4_·7H_2_O, 2.0 mg L^−1^ CoCl_2_·6H_2_O, 1.6 mg L^−1^ MnSO_4_·4H_2_O and 1.4 mg L^−1^ ZnSO_4_·7H_2_O. The batch composition for experiments with molasses and yeast-based medium was 20.0 g L^−1^ (NH_4_)_2_SO_4_, 30.0 g L^−1^ of sugars from sugarcane molasses and 20.0 g L^−1^ of an yeast-derived organic nitrogen source (yeast extract, YEPD-grown yeast or molasses-grown yeast preparations). The batch composition for fermentations with the molasses and ammonium sulfate medium was 30.0 g L^−1^ (NH_4_)_2_SO_4_ and 40.0 g L^−1^ of sugars from sugarcane molasses. In all fermentations, 1.0 mL L^−1^ of J647 antifoam (Struktol, Stow, USA) was added. 2 M phosphoric acid and 10% ammonia solutions were used for pH control. Aeration, dissolved oxygen (DO), pH and temperature parameters were set and maintained as described by Ellilä and colleagues [26]. In the synthetic medium fermentations, a solution containing 300 g L^−1^ of glucose and 200 g L^−1^ of Lactose was fed from 40 to 168 h at a rate of 1.0 g L^−1^ of sugar per hour. In all other experiments with Br_TrR03, sugarcane molasses was fed from 44 to 168 h at a rate of 1.0 g L^−1^ of total sugars from sugarcane molasses per hour. For experiments using the molasses-grown yeast preparation, the feed was extended to 336 h. In the bioreactor fermentations with Br_TrR02 and Br_TrR02B, (acid-inverted) sugarcane molasses was fed from 48 to 310 h at a rate of 0.65 g L^−1^ of total sugarcane molasses sugars per hour. Feeding was started when a reduction in growth by the fungus was observed. This was indirectly monitored through the volume of ammonium hydroxide solution required to maintain a stable pH. Initial batch volumes of 1.0 L were used, and the bioreactors were inoculated with 100 mL of a 3–7 days old shake flask preculture of the same media composition as the fermentation batch medium. Samples were withdrawn at regular intervals, centrifuged at 21,000×*g* for 10 min and the supernatants were stored at − 20 °C for analysis. The last point of the fermentations with RUT-C30 and Br_TrR03 on inducer-rich medium and Br_TrR03 with the MMGY medium were used for enzyme activity and saccharification assays. A non-centrifuged sample (whole broth) from Br_TrR03 with the MMGY medium was also collected and frozen for the saccharification assay. Due to its low protein concentration, the sample from the RUT-C30 fermentation with the inducer-rich medium was concentrated using a Vivaspin Turbo 15, 10,000 MWCO column prior to its use in saccharification assays.

### Enzyme and saccharification assays

The protein concentration and enzymatic activities of collected fermentation samples were determined as previously described [26]. In short, proteins were precipitated with ice-cold acetone and then quantified using the BioRad (Hercules, USA) DC protein assay kit, which is based on the method of Lowry [79]. Bovine Serum Albumin (BSA) was used as a standard. Filter paper activity (FPase) was measured using the standard method described by Ghose [80]. The β-glucosidase and β-xylosidase enzymatic activities were measured using the substrates 4-nitrophenyl-β-d-glucopyranoside (pNPG) and 4-nitrophenyl-β-d-xylopyranoside (pNPX), respectively. Reactions were carried out in 50 mM citrate buffer, pH 5.0, for 10 min at 50 °C and terminated with one volume of 1 M Na_2_CO_3_. In order to quantify the released 4-nitrophenol (pNP), absorbance was measured at 405 nm. Standard curves were prepared with pNP solutions of known concentrations. Cellobiohydrolase activity was measured with the 4-methylumbelliferyl-β-d-lactopyranoside (MUL) substrate. MUL is mainly hydrolyzed by *T. reesei’*s cellobiohydrolase I (CBHI) enzyme. The action of β-glucosidase (βG) and endoglucanase I (EGI) account for no more than 15% of measured activity [81]. Following incubation at 50 °C for 10 min, reactions were stopped by the addition of 1 volume of 1 M Na_2_CO_3_. Quantification of released 4-methylumbelliferone (MU) was accomplished by measuring fluorescence at 445 nm with an excitation wavelength of 380 nm. Endoglucanase activity was measured using carboxymethylcellulose (CMC), while xylanase activity was measured using beechwood xylan as a substrate. Both reactions were carried out in 50 mM citrate buffer, pH 5.0, at 50 °C for 5 min (xylanase) or 10 min (endoglucanase) and stopped by the addition of 1.5 volumes of 3,5-dinitrosalicylic acid (DNS) reagent, followed by heating at 95 °C for 5 min. Absorbance was measured at 540 nm in order to quantify the released reducing sugars. Pure glucose or xylose solutions were used as standards. All substrates and standards used in the enzyme assays were acquired from Sigma.

Saccharification reactions were performed in 50 mL Falcon tubes using a FINEPCR combi-D24 hybridization incubator (Gunpo-si, South Korea) set to maximum rotation (level 9) and 50 °C temperature. Pretreated sugarcane straw was provided by GranBio (São Miguel dos Campos, Brazil). This material was pretreated using proprietary technology at GranBio’s lignocellulosic ethanol factory BioFlex I. The substrate pH was adjusted to 5.0 with 10 M NaOH. Each hydrolysis reaction was set up as follows: total mass of 20.0 g, solid content of 20%, enzymes at a final concentration of 10 mg of protein/g of dry biomass, NaN_3_ to a final concentration of 0.02% and citrate buffer pH 5.0 to a final concentration of 100 mM. To ensure homogeneous reactions, two inox metal spheres (3.5 mm diameter and 1 g weight each) were inserted into each Falcon tube. A blank reaction was set up by substituting the added enzyme volume for dH_2_O. Following an incubation period of 72 h, 1 g of material from each tube was withdrawn, diluted in 9 mL of deionized water in a 15 mL Falcon tube, mixed thoroughly and centrifuged for 10 min at 2880×*g*. A 1.5 mL sample of the supernatant was transferred to a 2 ml Eppendorf tube, boiled for 10 min and then stored at − 20 °C until analysis. Sugars were quantified by HPLC (Additional file 1: Materials and methods). Sugar concentrations detected in the blank reaction were subtracted from the values obtained for the enzyme-containing reactions.

One-way analyses of variance (ANOVA) with post hoc Tukey HSD tests were conducted to compare the mean values of enzymatic activities, protein and sugar concentrations detected in the conducted assays.

## Supplementary information

**Additional file 1: Figure S1.** Sucrose consumption in shake flask cultivations. **Figure S2.** Extracellular protein titers in bioreactor cultivations. **Figure S3.** Target specific CRISPR/Cas9 plasmid map. **Figure S4.** Strategy for PCR verification of transformants. **Figures S5, S6 and S7**. PCR verification of transformants.** Table S1.** Oligonucleotides used in the study. Additional material and methods.

## Data Availability

The datasets used and/or analyzed during the current study are available from the corresponding author on reasonable request.
