# Supplementary material for: Rational engineering of the Trichoderma reesei RUT-C30 strain into an industrially relevant platform for cellulase production
Source: Biotechnol Biofuels. 2020 May 22;13:93. doi: 10.1186/s13068-020-01732-w (PMC7243233; doi:10.1186/s13068-020-01732-w)
Supplement: Supplementary file 1 — Additional file 1: Figure S1. Sucrose consumption in shake flask cultivations. Figure S2. Extracellular protein titers in bioreactor cultivations. Figure S3. Target specific CRISPR/Cas9 plasmid map. Figure S4. Strategy for PCR verification of transformants. Figures S5, S6 and S7. PCR verification of transformants. Table S1. Oligonucleotides used in the study. Additional material and methods. [file 13068_2020_1732_MOESM1_ESM.docx]

**Rational engineering of the *Trichoderma reesei* RUT-C30 strain into an industrially relevant platform for cellulase production**

Lucas Miranda Fonseca^#^, Lucas Salera Parreiras^#,*^ and Mario Tyago Murakami^*^

Brazilian Biorenewables National Laboratory, Brazilian Center for Research in Energy and Materials, Campinas, São Paulo, 13083-100, Brazil.

^#^Both authors contributed equally to this work.

^*^Correspondence should be addressed to L.S.P. ([lucas.parreiras@lnbr.cnpem.br](mailto:lucas.parreiras@lnbr.cnpem.br)) and M.T.M. ([mario.murakami@lnbr.cnpem.br](mailto:mario.murakami@lnbr.cnpem.br)).

**

**

**Figure S1: Sucrose consumption in shake flask cultivations.** Fungal strains were grown in Erlenmeyer flasks with medium containing 50 g L^-1^ of sucrose. Values indicate sucrose concentrations at the time of inoculation and after 5 days of cultivation. Different capital letters above the bars indicate cultures with different sucrose concentrations (by the Tukey HSD test with 95% simultaneous confidence intervals). Error bars, ± 1 standard deviation of the mean (n = 3).

**

**

**Figure S2: Extracellular protein titers in bioreactor cultivations.** Production of extracellular protein by Br_TrR02 (IHEM_5652) and Br_TrR02B (ATCC_56765) strains when cultivated in bioreactors under fed-batch mode with the MMGY medium (containing acid-inverted molasses).

**Figure S3:** Target specific CRISPR/Cas9 plasmid map.


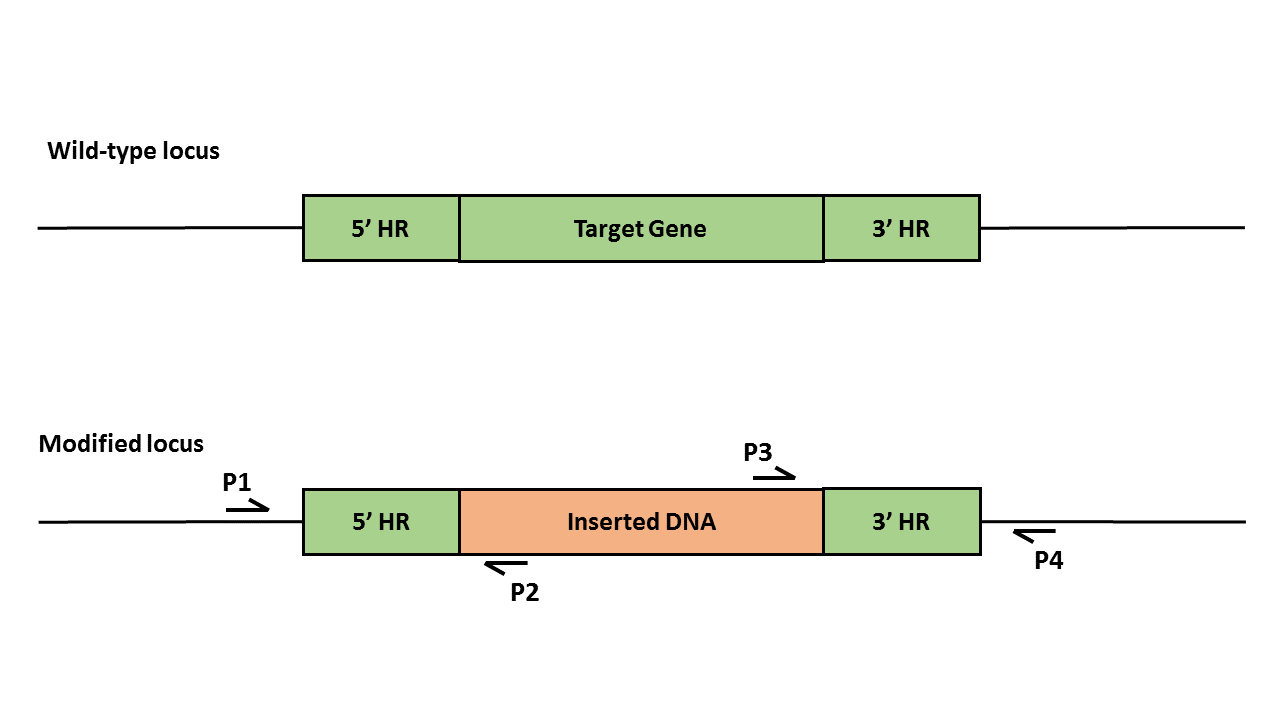


**Figure S4: Strategy for PCR verification of transformants.** The correct insertion of donor DNA cassettes in the fungal genome and the absence of heterokaryons was verified through 3 sets of PCR reactions conducted for each transformation event. Primers were designed to anneal to unique locations outside of homology arm regions (HR), both up (P1) and downstream (P4) of the targeted genomic regions and internally to donor DNA cassettes (P2 and P3). All PCR amplifications were performed with Phusion High Fidelity DNA polymerase following the manufacturer instructions. PCR reactions with a given set of primers were performed simultaneously and under the same conditions (including extension times) for all tested gDNA/isolates. The wild-type RUT-C30 gDNA was included as a control. The absence of heterokaryons was verified through the amplification of the genomic regions targeted by the donor DNA cassettes with primers P1 and P4. In all cases the sequences of donor DNA cassettes to be inserted were considerably longer than the DNA sequences to be excised, favoring the amplification of the wild-type sequence if present. The correct insertion of donor DNA cassettes was verified by combining primers that anneal outside the homology arm regions (HR) with primers that anneal internally to the donor DNA cassettes (P1 + P2 and P3 + P4). PCR products were run on 1% agarose gels. The 1Kb plus DNA molecular weight marker (Invitrogen) was included in the left-hand lane for each run. Agarose gels were stained with an ethidium bromide solution (0.5 mg ml-^1^) for approximately 30 min and then photographed with an UV transilluminator.


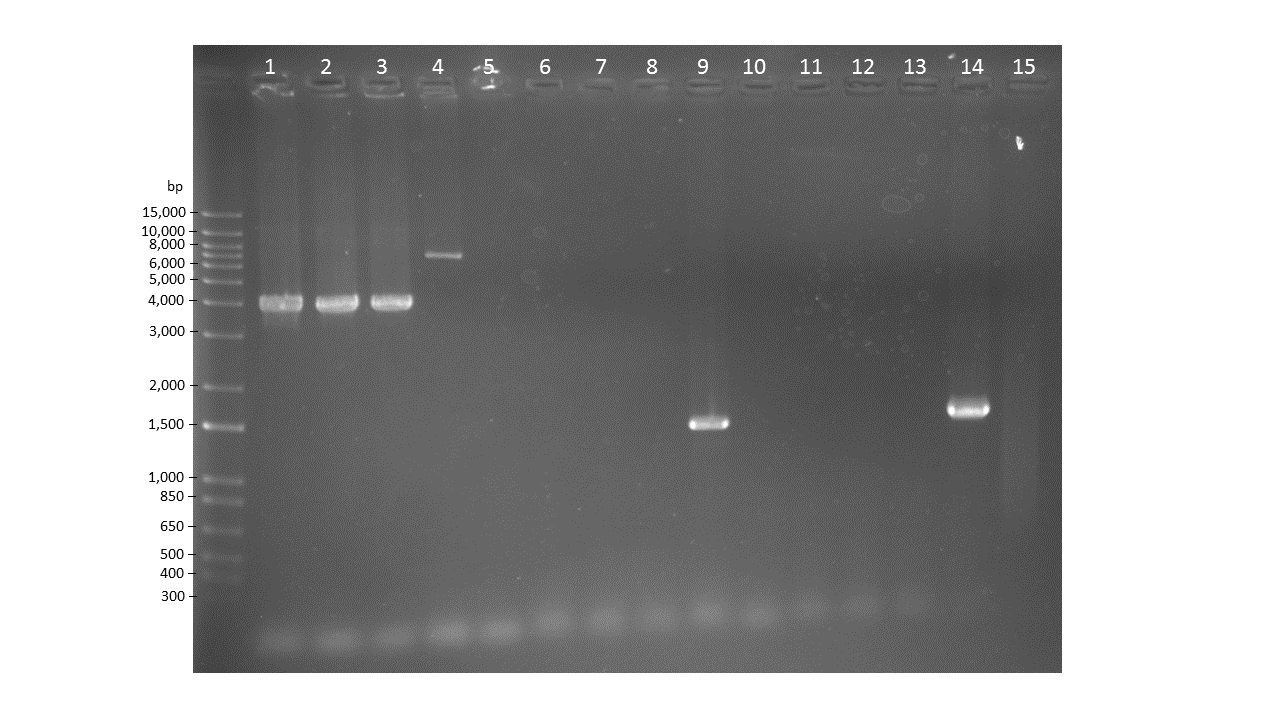


| **Reaction #** | **gDNA template** | **Primer set** | **Expected PCR product (bp)** |
| --- | --- | --- | --- |
| **1** | RUT-C30 | pep1TrH1-317FOR (P1)  and  pep1TrH2+320REV (P4) | 3928 |
| **2** | Br_TrR01 |  |  |
| **3** | Br_TrR02 |  |  |
| **4** | Br_TrR03 |  | 6876 |
| **5** | none |  | --- |
| **6** | RUT-C30 | pep1TrH1-317FOR (P1)  and  suc1An+168REV (P2) | --- |
| **7** | Br_TrR01 |  |  |
| **8** | Br_TrR02 |  |  |
| **9** | Br_TrR03 |  | 1479 |
| **10** | none |  | --- |
| **11** | RUT-C30 | suc1An-237FOR (P3)  and  pep1TrH2+320REV (P4) | --- |
| **12** | Br_TrR01 |  |  |
| **13** | Br_TrR02 |  |  |
| **14** | Br_TrR03 |  | 1549 |
| **15** | none |  | --- |

**Figure S5: PCR verification of transformants.** PCR verification of the simultaneous insertion of the *suc1* gene from *A. niger* and deletion of the *pep1* gene.


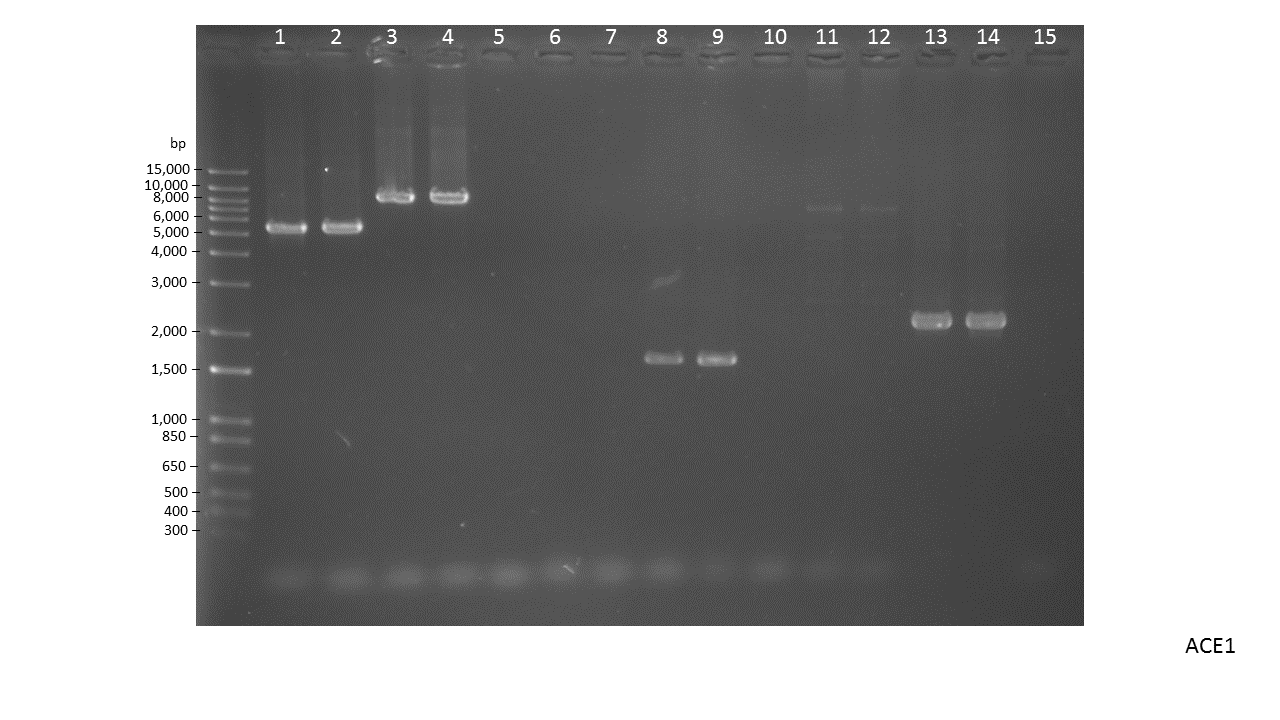


| **Reaction #** | **gDNA template** | **Primer set** | **Expected PCR product (bp)** |
| --- | --- | --- | --- |
| **1** | RUT-C30 | ace1TrH1-517FOR (P1)  and  ace1TrH2+497REV (P4) | 5400 |
| **2** | Br_TrR01 |  |  |
| **3** | Br_TrR02 |  | 8279 |
| **4** | Br_TrR03 |  |  |
| **5** | none |  | --- |
| **6** | RUT-C30 | ace1TrH1-517FOR (P1)  and  Pdc1p-1157REV (P2) | --- |
| **7** | Br_TrR01 |  |  |
| **8** | Br_TrR02 |  | 1651 |
| **9** | Br_TrR03 |  |  |
| **10** | none |  | --- |
| **11** | RUT-C30 | Pdc1t+314FOR (P3)  and  ace1TrH2+497REV (P4) | --- |
| **12** | Br_TrR01 |  |  |
| **13** | Br_TrR02 |  | 2227 |
| **14** | Br_TrR03 |  |  |
| **15** | none |  | --- |

**Figure S6: PCR verification of transformants.** PCR verification of the simultaneous insertion of the *xyr1*V821F allele and deletion of the *ace1* gene.


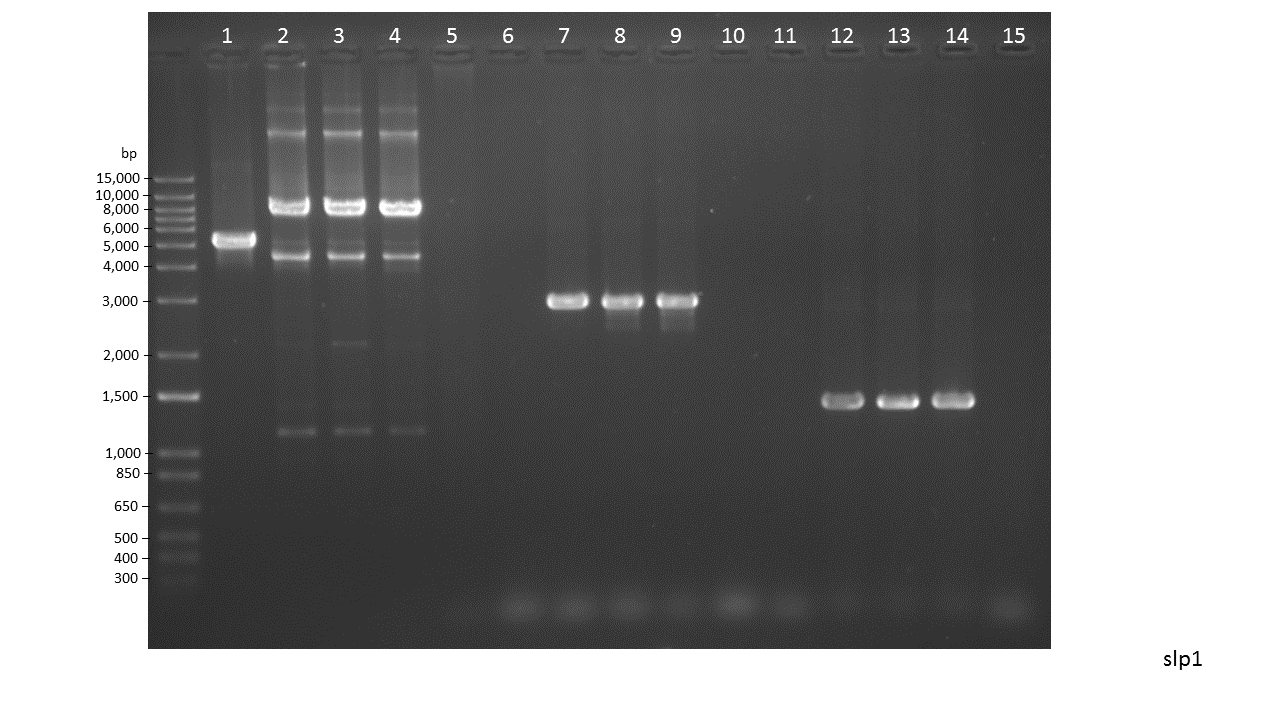


| **Reaction #** | **gDNA template** | **Primer set** | **Expected PCR product (bp)** |
| --- | --- | --- | --- |
| **1** | RUT-C30 | slp1TrH1-170FOR (P1)  and  slp1TrH2+227REV (P4) | 5153 |
| **2** | Br_TrR01 |  | 7826 |
| **3** | Br_TrR02 |  |  |
| **4** | Br_TrR03 |  |  |
| **5** | none |  | --- |
| **6** | RUT-C30 | slp1TrH1-170FOR (P1)  and  cel3aRe+224REV (P2) | --- |
| **7** | Br_TrR01 |  | 2894 |
| **8** | Br_TrR02 |  |  |
| **9** | Br_TrR03 |  |  |
| **10** | none |  | --- |
| **11** | RUT-C30 | xyn1tTr-172FOR (P3)  and  slp1TrH2+227REV (P4) | --- |
| **12** | Br_TrR01 |  | 1382 |
| **13** | Br_TrR02 |  |  |
| **14** | Br_TrR03 |  |  |
| **15** | none |  | --- |

**Figure S7: PCR verification of transformants.** PCR verification of the simultaneous insertion of the *cel3a* gene from *T. emersonii* and deletion of the *slp1* gene.

**Table S1: Oligonucleotides used in the study for plasmid assembly and PCR verification of transformants.**

|  | **Oligonucleotide** | **Sequence (5' -3')** | **Purpose** |
| --- | --- | --- | --- |
| 1 | KanMXFOR_pRS315 | TTAACCCTCACTAAAGGGAACAAAAGCTGGGTACCGGAGTTTTGACCATCAAAGAAGGTTAATG | Amplification of kanMX cassette |
| 2 | KanMXREV | CTTATAATACAGTTTTATAACTTCGTA | Amplification of kanMX cassette |
| 3 | pRS315_Apa1FOR | CCGGTACCCAGCTTTTGTT | Ampllification of pRS315 plasmid backbone |
| 4 | pSH315_BspREV | TGAAATTCATAATAGAAACGACACG | Ampllification of pRS315 plasmid backbone |
| 5* | ACE1TrProtInsRibo | AGATTCTGTACATCCTCCCTGATGAGTCCGTGAGGACGAAACGAGTAAGCTCGTC**GGAGGAAGGCGTAGACACGT**GTTTTAGAGCTAGAAATAGCAAG | Assembly of gRNA for ace1 gene disruption |
| 6 | HHRiboACE1TrprotFOR | AGATTCTGTACATCCTCCCTGA | Assembly of gRNA for ace1 gene disruption |
| 7* | Pep1TrProtInsRibo | AGCTTCTGTACAAGCTCCCTGATGAGTCCGTGAGGACGAAACGAGTAAGCTCGTC**GGAGCTAAAGACCCACAGGT**GTTTTAGAGCTAGAAATAGCAAG | Assembly of gRNA for pep1 gene disruption |
| 8 | HHRiboPep1TrprotFOR | AGCTTCTGTACAAGCTCCCTGA | Assembly of gRNA for pep1 gene disruption |
| 9 | HHRiboSLP1TrprotFOR | AGCTTCTGTACAGCTGTCCTGA | Assembly of gRNA for slp1 gene disruption |
| 10* | Slp1TrProtInsRibo | AGCTTCTGTACAGCTGTCCTGATGAGTCCGTGAGGACGAAACGAGTAAGCTCGTC**GACAGCCTCAATCTCGGGGA**GTTTTAGAGCTAGAAATAGCAAG | Assembly of gRNA for slp1 gene disruption |
| 11 | HDVRibo_BSP1407IREV | TCAATGTACAGTCCCATTCGCCATGCCG | Assembly of gRNAs for insertion in the pTRCas9gRNA1 vector |
| 12 | HDVRibo_BSP1407I | TCAATGTACAGTCCCATTCGCCATGCCGAAGCATGTTGCCCAGCCGGCGCCAGCGAGGAGGCTGGGACCATGCCGGCCAAAAGCACCGACTCGGTGCCACTTTTTCAAGTTGATAACGGACTAGCCTTATTTTAACTTGCTATTTCTAGCTCTAAAAC | Assembly of gRNAs for insertion in the pTRCas9gRNA1 vector |

***** Highlighted sequences correspond to 20-nt protospacers designed to target the respective *T. reesei* genes.

|  | **Oligonucleotide** | **Sequence (5' -3')** | **Purpose** |
| --- | --- | --- | --- |
| 13 | pep1TrH2REV_NotIpRS315 | TCCATTTTGTAATTTCGTGTCGTTTCTATTATGAATTTCAGCGGCCGCCTCCCCTGTCAATTCCCTTA | Assembly of pRS315_AnSuc1:pep1Δ plasmid |
| 14 | pep1TrH2FOR_Suc1An | GTGGTGGGTTGGACGCGAAGCACTTCATCCAGCTCCTCGTGGCGATGGTGGACTTGTTTA | Assembly of pRS315_AnSuc1:pep1Δ plasmid |
| 15 | Suc1tAnREV | ACGAGGAGCTGGATGAAGTG | Assembly of pRS315_AnSuc1:pep1Δ plasmid |
| 16 | pep1TrH1REV_Suc1An | TTGGCATTGCCAACGAGCCCCATGCACGAGTGAAGTAAAGTTGAATATCGGAGAAGGTTGC | Assembly of pRS315_AnSuc1:pep1Δ plasmid |
| 17 | Suc1pAnFOR | CTTTACTTCACTCGTGCATGG | Assembly of pRS315_AnSuc1:pep1Δ plasmid |
| 18 | pep1TrH1FOR_NotIKanMX | ATAATGTATGCTATACGAAGTTATAAAACTGTATTATAAGGCGGCCGCCTGGTGACTGGCCGTCTG | Assembly of pRS315_AnSuc1:pep1Δ plasmid |
| 19 | PDC1pTrFOR_ACE1H1 | GGACTCGTGTGGCCATATCTCATACATCTTCCCCACCCACAGGGAGCATTCTTCGACTTG | Assembly of pRS315_Ppdc1_xyr1V821F_Tpdc1:ace1Δ plasmid |
| 20 | PDC1pTrREV_Xyr1 | TGTCGGGGTAGGCAGAATAGCGACGGAGAGGATTGGACAACATGATTGTGCTGTAGCTGC | Assembly of pRS315_Ppdc1_xyr1V821F_Tpdc1:ace1Δ plasmid |
| 21 | Xyr1TrFOR | TTGTCCAATCCTCTCCGTCGCTAT | Assembly of pRS315_Ppdc1_xyr1V821F_Tpdc1:ace1Δ plasmid |
| 22 | Xyr1TrMREV | CGGCAGCCGAGAACGCGTGGCTCGTC | Assembly of pRS315_Ppdc1_xyr1V821F_Tpdc1:ace1Δ plasmid |
| 23 | Xy1rTrMFOR | ACGCGTTCTCGGCTGCCGAAGCTATTAG | Assembly of pRS315_Ppdc1_xyr1V821F_Tpdc1:ace1Δ plasmid |
|  | **Oligonucleotide** | **Sequence (5' -3')** | **Purpose** |
| 24 | PDC1tTRFOR_Xyr1 | CGATGGACTGGTAACGGAACCGGTCTGGCCCTCTAAGGAGGCATGAAGTCTGACCGGGTA | Assembly of pRS315_Ppdc1_xyr1V821F_Tpdc1:ace1Δ plasmid |
| 25 | Xyr1TrREV | CTCCTTAGAGGGCCAGACC | Assembly of pRS315_Ppdc1_xyr1V821F_Tpdc1:ace1Δ plasmid |
| 26 | PDC1tTrREV | CCTCGATGTCTTCCTCTTCG | Assembly of pRS315_Ppdc1_xyr1V821F_Tpdc1:ace1Δ plasmid |
| 27 | ACE1TrH1FOR_KanMX | ATAATGTATGCTATACGAAGTTATAAAACTGTATTATAAGAGCAGGGATACGCCTCCTC | Assembly of pRS315_Ppdc1_xyr1V821F_Tpdc1:ace1Δ plasmid |
| 28 | ACE1TrH1REV | GTGGGTGGGGAAGATGTATG | Assembly of pRS315_Ppdc1_xyr1V821F_Tpdc1:ace1Δ plasmid |
| 29 | PDC1pTrFOR_ACE1H1 | GGACTCGTGTGGCCATATCTCATACATCTTCCCCACCCACAGGGAGCATTCTTCGACTTG | Assembly of pRS315_Ppdc1_xyr1V821F_Tpdc1:ace1Δ plasmid |
| 30 | PDC1pTrREV_Xyr1 | TGTCGGGGTAGGCAGAATAGCGACGGAGAGGATTGGACAACATGATTGTGCTGTAGCTGC | Assembly of pRS315_Ppdc1_xyr1V821F_Tpdc1:ace1Δ plasmid |
| 31 | AcTrH2FOR_Pdc1t | CACCACCCGGCCGACGGAGCCGAAGAGGAAGACATCGAGGCAAGATGCTGCAAACGCTTA | Assembly of pRS315_Ppdc1_xyr1V821F_Tpdc1:ace1Δ plasmid |

|  | **Oligonucleotide** | **Sequence (5' -3')** | **Purpose** |
| --- | --- | --- | --- |
| 32 | ACE1TrH2REV_pRS315 | TCCATTTTGTAATTTCGTGTCGTTTCTATTATGAATTTCAAATTTCTTCCCCTCCAAACC | Assembly of pRS315_Ppdc1_xyr1V821F_Tpdc1:ace1Δ plasmid |
| 33 | slp1TrFORnheI_pRS315 | ATAATGTATGCTATACGAAGTTATAAAACTGTATTATAAGGCTAGCAATGCTGTGCACTTGTCTCG | Assembly of the pRS315_Pxyn1_ReCel3a_Txyn1:slp1Δ plasmid |
| 34 | slp1TrREV_xyn1p | TCAATCTGCCAGCCAACGAAATATGCTGTGATTAAGTCTTTCACACTCTATCCCGCTTGTC | Assembly of the pRS315_Pxyn1_ReCel3a_Txyn1:slp1Δ plasmid |
| 35 | Xyn1pTrFOR | AAGACTTAATCACAGCATATTTCGTT | Assembly of the pRS315_Pxyn1_ReCel3a_Txyn1:slp1Δ plasmid |
| 36 | Xyn1pTrREV_Cel3aRe | CCGCAAGGGCGGCGACCTTGAGCAACCCGTTCCTCATCGTGATGATTATTTGTGCGTGTTTTC | Assembly of the pRS315_Pxyn1_ReCel3a_Txyn1:slp1Δ plasmid |
| 37 | Cel3aReFOR | ACGATGAGGAACGGGTTG | Assembly of the pRS315_Pxyn1_ReCel3a_Txyn1:slp1Δ plasmid |
| 38 | Xyn1tTrFOR_Cel3aRe | GCCTTTGCAGGCACCCCTTAAGCCATATCCTGGAATCTAATCTGTTGATGTTGACTTGGAGTG | Assembly of the pRS315_Pxyn1_ReCel3a_Txyn1:slp1Δ plasmid |
| 39 | Cel3aReREV | TTAGATTCCAGGATATGGCTTA | Assembly of the pRS315_Pxyn1_ReCel3a_Txyn1:slp1Δ plasmid |
| 40 | slp1TrH2FOR_Xyn1t | CTCGGAAGAGATCGAGGACATTGTTGATCCCATACTGGGCAGATGTAAGAGGGTTTCTTGAGG | Assembly of the pRS315_Pxyn1_ReCel3a_Txyn1:slp1Δ plasmid |
| 41 | Xyn1tTrREV | GCCCAGTATGGGATCAACAA | Assembly of the pRS315_Pxyn1_ReCel3a_Txyn1:slp1Δ plasmid |
| 42 | slp1H2REVnheI_pRS315 | TCCATTTTGTAATTTCGTGTCGTTTCTATTATGAATTTCAGCTAGCGACGGCACCTGGTTGGAT | Assembly of the pRS315_Pxyn1_ReCel3a_Txyn1:slp1Δ plasmid |
|  | **Oligonucleotide** | **Sequence (5' -3')** | **Purpose** |
| 43 | Pep1TrH1FOR | CTGGTGACTGGCCGTCTG | PCR amplification of the AnSuc1:pep1Δ cassette |
| 44 | Pep1TrH2REV | CTCCCCTGTCAATTCCCTTA | PCR amplification of the AnSuc1:pep1Δ cassette |
| 45 | ACE1TrH1FOR | AGCAGGGATACGCCTCCTC | PCR amplification of the Ppdc1_xyr1V821F_Tpdc1:ace1Δ cassette |
| 46 | ACE1TrH2REV | AATTTCTTCCCCTCCAAACC | PCR amplification of the Ppdc1_xyr1V821F_Tpdc1:ace1Δ cassette |
| 47 | slp1TrH1FOR | AATGCTGTGCACTTGTCTCG | PCR amplification of the Pxyn1_ReCel3a_Txyn1:slp1Δ cassette |
| 48 | slp1TrH2REV | GACGGCACCTGGTTGGAT | PCR amplification of the Pxyn1_ReCel3a_Txyn1:slp1Δ cassette |
| 49 | pUC19OriREV_BSAhph | GTAGTCGGTCTCCCCAGCATTAATGAATCGGCCAAC | pTrCas9gRNA1 plasmid assembly |
| 50 | PDC1tTrREV_BSAeno1p | GTAGTCGGTCTCGTAGCTGGCGTCTTCCATCTT | pTrCas9gRNA1 plasmid assembly |
| 51 | Xyn1tFOR_BSAbsp | GTAGTCGGTCTCCCATTGACTTGGAGTGGATGAGG | pTrCas9gRNA1 plasmid assembly |
| 52 | Xyn1tREV_BSAhph | GTAGTCGGTCTCCAGAGGATCTAGCGGGAGGATT | pTrCas9gRNA1 plasmid assembly |
| 53 | hphFOR_BSAxyn1t | GTAGTCGGTCTCGCTCTGACCGGTGACTCTTTCTGG | pTrCas9gRNA1 plasmid assembly |
| 54 | Pdc1pTRFOR_BSA_NotI | TCAGGTCTCAGGCCGCATGCACATGTACGATTGG | pTrCas9gRNA1 plasmid assembly |
| 55 | pUC19REV_BSA_NotI | TTAGGTCTCAGGCCAGGCCACGAAAGGGCCTCGTGATAC | pTrCas9gRNA1 plasmid assembly |
| 56 | Eno1pFOR_BSApdc1t | AGTGGTCTCAGCTAGATTCCGTCCTGGATTGC | pTrCas9gRNA1 plasmid assembly |

|  | **Oligonucleotide** | **Sequence (5' -3')** | **Purpose** |
| --- | --- | --- | --- |
| 57 | Eno1pREVBSP_BSAxyn1t | GTAGGTCTCTAATGTACAGAAGCTATTTCAGGTGGCTGGA | pTrCas9gRNA1 plasmid assembly |
| 58 | hphREV_BSApUC19 | ATGCGGTCTCGCTGGCAAGTGTACCTGTGCATTCTGG | pTrCas9gRNA1 plasmid assembly |
| 59 | hph-954FOR | ATATGCTCCGCATTGGTCTT | pTrCas9gRNA1 plasmid assembly verification |
| 60 | hph+267REV | GCTATACACCCCTGGCTTCA | pTrCas9gRNA1 plasmid assembly verification |
| 61 | pUC19ORI-334REV | GGGAAACGCCTGGTATCTTT | pTrCas9gRNA1 plasmid assembly verification |
| 62 | pUC19ampR-158FOR | ATTGTCTCATGAGCGGATACA | pTrCas9gRNA1 plasmid assembly verification |
| 63 | AMA1-280FOR | TGTTAACCTCTCGGGCTCTG | pTrCas9gRNA1 plasmid assembly verification |
| 64 | Eno1p-212FOR | GGCAAGTGGCCCAATTATTA | pTrCas9gRNA1 plasmid assembly verification |
| 65 | Xyn11BtTR+145FOR | CCTTTGACGCTGTATGCTCA | pTrCas9gRNA1 plasmid assembly verification |
| 66 | Xyn1t+318REV | TGGCTTTCGGGGTATATGAA | pTrCas9gRNA1 plasmid assembly verification |
| 67 | Suc1tAn-237FOR | GGGTCAATGTCGTAGCAGGT | Verification of the AnSuc1 cassette insertion at the pep1 locus |
| 68 | Suc1pAn+168REV | CTGGAAGGACCTTGTCGTTG | Verification of the AnSuc1 cassette insertion at the pep1 locus |
| 69 | pep1TrH1-317FOR | GACCAGCCCCAGAATCAC | Verification of the AnSuc1 cassette insertion at the pep1 locus |
| 70 | pep1TrH2+320REV | GACGGCGTTGATGTACAAGTT | Verification of the AnSuc1 cassette insertion at the pep1 locus |
| 71 | ACE1H1Tr-517FOR | TCAACGTCCAGTCCGTGTAA | Verification of the Ppdc1_xyr1V821F_Tpdc1 cassette insertion at the ace1 locus |
|  | **Oligonucleotide** | **Sequence (5' -3')** | **Purpose** |
| 72 | ACE1TrH2+497REV | AGCTTCTGAAAACCCCGATG | Verification of the Ppdc1_xyr1V821F_Tpdc1 cassette insertion at the ace1 locus |
| 73 | PDC1pTR-1157REV | CTAATTGCCCCTGTTTCCAA | Verification of the Ppdc1_xyr1V821F_Tpdc1 cassette insertion in the ace1 locus and of the pTrCas9gRNA1 assembly |
| 74 | PDC1tTR+314FOR | CTCGGGTACCTGTTCTTTCC | Verification of the Ppdc1_xyr1V821F_Tpdc1 cassette insertion in the ace1 locus and of the pTrCas9gRNA1 assembly |
| 75 | slp1H1-170FOR | TTCCTTTGGCCAATTCTTTG | Verification of the Pxyn1_ReCel3a_Txyn1 cassette insertion at the slp1 locus |
| 76 | slp1H2+227REV | CCCGACTTTTTGGTGTCATT | Verification of the Pxyn1_ReCel3a_Txyn1 cassette insertion at the slp1 locus |
| 77 | Cel3aRe+224REV | ACTTGACGGCCTTCTCGTAG | Verification of the Pxyn1_ReCel3a_Txyn1 cassette insertion at the slp1 locus |
| 78 | Xyn1tTr-172FOR | ACCAGGCGCAGAGAACATC | Verification of the Pxyn1_ReCel3a_Txyn1 cassette insertion at the slp1 locus |

**Additional material and methods**

**HPLC analyses**

Glucose and xylose concentrations in enzymatic hydrolysis samples were determined by high-performance liquid chromatography. Analyses were conducted with an Agilent (Santa Clara, USA) 1260 Infinity equipment with a refractive index detector, Aminex HPX 87H (BioRad) analytical column (300 mm x 7.8 mm) and pre-column (30 mm x 4.6 mm) at 35 °C, analysis flow of 0.6 mL min^-1^ and isocratic elution with 5 mM sulfuric acid. Sucrose, glucose and fructose quantifications in sugarcane molasses samples were performed with a Dionex (Sunnyvale, USA) Ultimate 3000 equipment with a refractive index detector, Aminex HPX-87P analytical column (300 mm x 7.8 mm) and pre-column (30 mm x 4.6 mm), at 55 °C and analysis flow of 0.5 mL min^-1^ and isocratic elution with ultrapure water. All samples were homogenized, centrifuged and filtered through a 0.22 µm-13 mm diameter Millex syringe filter (Sigma). The quantification of analytical results was performed by external calibration with the appropriate standard solutions.

**Sucrose quantification in shake flask experiments**

Sucrose concentration in shake flask experiments was determined by acid-hydrolysis followed by DNS quantification. 100 uL of 2M hydrochloric acid were added to 100 ul of culture supernatant properly diluted in water. The reaction was heated at 70 °C for 10 min and then allowed to cool. The pH of the solution was neutralized with the addition of 300uL of 1M NaOH and sugars were quantified with DNS, as described for the enzymatic assays.
